# A diet containing a nonfat dry milk matrix significantly alters systemic oxylipins and the endocannabinoid 2-arachidonoylglycerol (2-AG) in diet-induced obese mice

**DOI:** 10.1186/1743-7075-11-24

**Published:** 2014-05-30

**Authors:** Tamara N Dunn, Alison H Keenan, Anthony P Thomas, John W Newman, Sean H Adams

**Affiliations:** 1 Graduate Group in Nutritional Biology, University of California, Davis, USA; 2 Current address: Center for Computational and Integrative Biology, Massachusetts General Hospital, Boston, MA, USA; 3 Current Address: Larry L. Hillblom Islet Research Center, University of California, Los Angeles, David Geffen School of Medicine, Los Angeles, CA, USA; 4 Obesity & Metabolism Research Unit, USDA-Agricultural Research Service Western Human Nutrition Research Center, Davis, CA, USA; 5 Department of Nutrition, University of California, Davis, USA; 6 USDA-ARS Western Human Nutrition Research Center, 430 W. Health Sciences Dr, Davis, CA 95616, USA

## Abstract

**Background:**

Diets rich in dairy and/or calcium (Ca) have been associated with reductions in adiposity and inflammation, but the mechanisms underlying this remain to be fully elucidated. Oxylipins and endocannabinoids are bioactive lipids, which influence energy homeostasis, adipose function, insulin signaling, and inflammation. Our objective was to determine if these metabolites associate with metabolic and inflammatory phenotypes stemming from dietary Ca and dairy in diet induced obese mice.

**Methods:**

In one study, C57BL6/J mice were fed high fat diets (45% energy) with varying dietary matrices for 12 weeks: soy protein and Ca adequate (0.5%; CONTROL), soy protein and high Ca (1.5%; HighCa), or nonfat-dry-milk based high Ca (NFDM). In a second study, mice were pre-fattened for 12 weeks on the CONTROL high fat diet, and then fed one of three high fat diets for an additional 8 weeks: CONTROL, HighCa, or NFDM. In both studies, adiposity and associated metabolic and inflammatory outcomes were measured and a targeted lipidomics analysis was performed on plasma collected during the post-absorptive condition.

**Results:**

As reported previously, mice fed NFDM had less body fat and reduced mRNA markers of adipose inflammation (p < 0.05) than CONTROL mice despite greater cumulative energy intake. Moreover, NFDM fed mice lipid mediator profiles were distinct from CONTROL and HighCa mice. NFDM fed mice showed elevated plasma monoacylglycerols (6 – 46% increase from CONTROL), including 2-arachidonoylglycerol (2-AG), and reduced fatty acid diols (8-75% decrease from CONTROL).

**Conclusions:**

Differences in specific plasma lipid mediator profiles reflect the metabolic and inflammatory phenotypes seen in NFDM feeding.

## Background

Obesity has been characterized as a state of chronic inflammation, displaying increased macrophage and adipocyte release of interleukins and inflammatory cytokines as well as macrophage accumulation in adipose tissue [1,2]. The presence of this low-grade systemic inflammation contributes to the pathogenesis of metabolic diseases (i.e. insulin resistance, diabetes, cardiovascular disease) [3,4]. While weight loss, through calorie reduction, can reduce obesity-associated inflammation [5], there is increasing interest in identifying foods and micronutrients that can dampen the inflammatory profile independent of weight. For instance, there is strong epidemiological evidence to suggest a beneficial effect of dairy consumption on adiposity and inflammation [6,7], but randomized trials in humans are inconclusive [8-10]. Differences in specific dietary components or dairy foods in these studies may be responsible for the disparity of results. Furthermore, it can be difficult to dissociate inflammatory outcomes from changes in adiposity, since the latter directly correlates with white adipose tissue (WAT) macrophage infiltration and inflammatory markers over a wide range, at least in mice [11]. Therefore, it is important to identify which components of dairy and by what mechanism dairy foods exert anti-inflammatory and anti-obesity effects.

One proposed mechanism by which dairy can improve inflammatory status is through dairy-derived calcium’s ability to suppress circulating levels of 1,25-dihydroxyviatmin D_3_ (calcitriol). *In vivo* evidence has shown increased dietary calcium, both with and without dairy, diminished inflammation and oxidative stress in genetically-modified obese animals, with the interpretive caveat that adiposity was also reduced under these conditions [12]. Increased dietary calcium dampens circulating calcitriol, and calcitriol has been shown to activate pro-inflammatory pathways by inducing reactive oxygen species generation and cytokine release in adipocytes and macrophages [13,14]. However, anti-inflammatory actions of calcitriol have also been reported in humans [15], and serum calcitriol has been reported to be significantly lower in obese verses non-obese human subjects [16]. In diet-induced obese (DIO) mice fed high calcium or high-calcium plus dairy, circulating calcitriol did not correlate with alterations in systemic or WAT inflammation [17,18]. Thus, changes in calcitriol due to dietary calcium cannot fully explain the apparent anti-inflammatory effects of dairy.

Non-calcium components in dairy foods could also play a role in obesity-associated inflammation and regulation of metabolism. Dairy bioactives such as lactalbumin, angiotensin-converting enzyme inhibitor and branched-chain amino acids may modify metabolic and other physiological functions [19-23]. Additionally, evidence indicates that dairy proteins may have beneficial effects on appetite in humans [24], glucose metabolism and muscle protein synthesis [25], as well as improve fasting lipid and insulin levels [26,27]. In animal models of obesity, inclusion of dairy or dairy-based proteins in the diet reduces weight gain and adiposity, even in the absence of lower energy intake. In some studies in DIO mice fed a high fat diet containing a dairy matrix (e.g., a calcium rich, non-fat dry milk (NFDM) protein and carbohydrate-based diet), energy intake was modestly increased, resulting in lower feed efficiency [17,18]. The etiology of these effects on food intake and energy balance remains to be ascertained.

Systemic energy balance is delicately regulated by multiple interactive systems, including a suite of endocannabinoids (eCBs) and oxylipins, low abundance signaling lipids present in the open circulation and tissues. To date, no attention has been given to the impact of a dairy-based diet on these lipid mediators, despite their roles in inflammation, food intake behaviors, thermogenesis, and a variety of other processes [28,29]. Oxylipins, oxygenated products of fatty acid metabolism that include the arachidonic acid-derived eicosanoids, have numerous bioactive properties including both pro- and anti-inflammatory effects [30], constrictive and dilatory vascular effects [31], and effects on insulin signaling [29,32]. The eCBs are lipid mediators that elicit effects on energy homeostasis and immune function through interactions with the cannabinoid type 1 and type 2 (CB1 and CB2) receptors and transient receptor potential cation channel 1 (TRPV1). eCBs may also bind and activate peroxisome proliferator-activated receptor γ (PPARγ), a transcription factor that regulates genes to promote lipid storage (and immunomodulation) [33]. The archetypical eCBs are the arachidonate-derived 2-archidonoylglycerol (2-AG) and N-arachidonoyl-ethanolamine (AEA). In addition to neuromodulatory effects on appetite, eCB signaling has been implicated in the pathogenesis of obesity by actions that promote lipogenesis in adipose and liver, and may also regulate insulin sensitivity [34-36].

Besides the effects of dietary long chain PUFAs on circulating oxylipins and eCBs concentrations [37,38], the impact of other nutrients on these systems is not known. Given the putative role of dairy and calcium in adiposity and inflammation, we hypothesized that these nutrients would impact signaling lipid profiles, and we tested this under conditions of both developing and pre-existing obesity. Using targeted quantitative metabolic profiling we performed a secondary analysis of archived plasma from a previous study investigating the effects of dairy and calcium on inflammatory phenotypes in a DIO mouse model [17,18]. As previously reported, animals fed a high fat diet rich in calcium in a NFDM-based matrix showed decreased adiposity, markedly reduced steatosis, and lower adipose inflammation in comparison to a high-calcium, soy protein-based high fat diet [17,18]. In the current study, signaling lipid profiling, gene expression, and gross morphometric parameters were used to describe phenotypes in high fat fed mice that best discriminated NFDM protein and carbohydrate-fed mice from animals fed soy protein/sucrose carbohydrate-based diets. We contend that the identified shifts in oxylipin and endocannabinoid tone may be associated with biochemical and physiological processes that drive the markedly different energy balance and inflammatory phenotype observed in with NFDM feeding in mice.

## Methods

### Animals

All animal protocols were approved by the University of California at Davis Institutional Animal Care and Use Committee according to Animal Welfare Act guidelines.

#### COHORT 1: developing obesity model

Details for Cohort 1 regarding housing conditions, diets, tissue/blood collection and other experimental variables have been previously published [17]. In brief, 5 week old C57BL/6 J weight-matched male mice were randomly assigned to one of three obesogenic diets containing 45% energy as fat (lard) for 12 weeks. This diet and 12 week timeframe were previously shown to elicit obesity along with increased WAT inflammation and insulin resistance in this model [11]. Treatment groups were: 1) soy protein- and sucrose carbohydrate based control (0.5% calcium, CONTROL), 2) soy and sucrose-based high-calcium (1.5% calcium, HighCa), or 3) high-calcium (1.5%) in the context of non-fat dry milk protein and carbohydrates (NFDM). Mice were given free access to food and water with body weight and food intake (plus spillage) measurements made every 2–3 d. At week 12, mice were briefly food deprived (ca. 4–6 hr into the light cycle) in the morning prior to being deeply anesthetized via isoflurane inhalation (3% in O_2_). After exposing the diaphragm, blood was collected by cardiac puncture using EDTA-treated syringes. Mice did not survive this procedure. Blood plasma was subsequently collected by centrifugation at 10,000 × g for 2 minutes and snap-frozen in liquid nitrogen. Retroperitoneal white adipose tissue (RP-WAT), liver, and nodose ganglia were excised and snap-frozen in liquid nitrogen. All tissues were stored at −80°C. A subsample of animals (n = 10/treatment group) was randomly selected for plasma and tissue analysis.

#### COHORT 2: pre-existing obesity model

Details for Cohort 2 regarding housing conditions, diets, and other variables have been previously published [18]. In brief, 5 week old C57BL/6 J male mice were fed the CONTROL diet for 12 weeks. After the 12 week, pre-fattening period, weight-matched DIO mice were randomly assigned to one of three macronutrient-matched high fat diets for an additional 8 weeks: 1) CONTROL, 2) HighCa, or 3) NFDM. At week 8 of intervention, mice were briefly food deprived prior to tissue and blood collection (described above). A subsample of animals (n = 10/treatment group) was randomly selected for plasma and tissue analysis.

### Plasma lipid mediator profiling

Calibrants and isotopically labeled surrogates were purchased from either Cayman Chemical (Ann Arbor, MI) or Larodan Fine Chemicals (Malmo, Sweden). Calibration solutions were prepared in 50/50 acetonitrile:methanol (v/v), ampuoled under dry nitrogen, and stored at -20°C until use. Analyte retention times, surrogates-associations, and tandem mass spectrometry parameters are provided in Additional file 1: Table S5. Oxylipins and eCBs were isolated by solid phase extraction and quantified by LC-MS/MS using modifications of published protocols [39,40]. Briefly, plasma aliquots (100 μL; n = 10/group) were first introduced to 60 mg Oasis HLB (Waters Corporation, Milford, MA) SPE cartridges reservoirs, enriched with deuterated oxylipin and eCB surrogates and antioxidants (BHT/EDTA), and diluted with 2 mL of 5% methanol/0.1% acetic acid. Samples were loaded by gravity, washed with 20% methanol/0.1% acetic acid and air dried. Loaded cartridges were then wetted with methanol and eluted with ethyl acetate into 6 μL glycerol, and the solvent was evaporated under vacuum. The resulting glycerol plug was stored at -80°C until reconstitution in methanol containing the internal standard 1-cyclohexyl-urido-3-dodecanoic acid (Sigma-Aldrich, St. Louis MO), and analyzed within 48 hrs to limit monoacylglycerol isomerization. Analytes were separated on a 2.1 × 150 mm, 1.7 μm Acquity BEH column on a Waters Acquity UPLC. Analytes were ionized by electrospray ionization and detected by multi-reaction monitoring on an API4000 QTRAP (AB-SCIEX, Foster City, CA). Oxylipins and NAEs/MAGs/LAAs were analyzed in independent injections and ionized in negative and positive modes, respectively. Surrogate recoveries for reported oxylipins and eCBS were acceptable and stable across all analytical batches. Isomerization of d8-2-AG was <5% for all measured samples, and both 1-AG and 2-AG were chromatographically resolved (Additional file 2: Figure S1). Analytes are not reported if the signal-to-noise ratio is <2, calculated concentrations are below the lowest calibrant, or apparent surrogate recoveries are below 40%, suggesting either significant loss or suppression of electrospray ionization. For a complete list of lipid analytes that were retained for multivariate analysis, see Additional file 3: Tables S1 and S3.

### Total RNA isolation and gene expression analyses

The expression of genes associated with oxylipin and eCB function was assessed in liver, RP-WAT, and nodose ganglia to provide a contextual interpretation to diet-associated differences in plasma mediator tone. RNA was isolated from RP-WAT fat pads and liver samples using a Ribopure kit (Applied Biosystems-AM1924) according to manufacturer's instructions. Nodose ganglia mRNA was extracted using micro-Ribopure filters (AB AM1931). RNA abundance was quantified using a NanoDrop ND-1000 Spectrophotometer (NanoDrop Technologies). cDNA was prepared, and 384-well quantitative PCR utilizing gene-specific Taqman® primers and FAM-MGB labeled probes (Assays-on-Demand®, Applied Biosystems) was conducted as previously described in detail [11], with the exception that Eukaryotic 18S (ABI) was used as the endogenous reference gene for all tissues. See Additional file 3: Tables S2 and S4 for a complete list of gene targets.

### Data analysis and statistics

Data treatment prior to analysis included missing value imputation if variable sets were >80% complete and normality transformation, as well as mean center/unit variance scaling prior to multivariate analyses [41]. Imputation was performed independently for each metabolite class using probabilistic principal components analysis [42]. Missing prostanoids, fatty acid alcohols, fatty acid diols, acyl ethanolamides and monoacyl glycerides were imputed independently. Data were then transformed to normality using the procedures of Box and Cox [43]. Data imputations, transformations, and multivariate analyses were performed using the Excel Add-In imDEV [44], which provides a graphical user interface to the R Statistical Computing Environment [45].

Mean differences in plasma lipids and tissue gene expression were evaluated in Graphpad Prism V.6, after outlier exclusion with the false discovery rate based ROUT Test. If data passed the D’Agostino and Pearson’s omnibus test for normality, then dietary treatment differences were assessed by one-way ANOVA with a Tukey post-hoc test. If data were not normal, the Kruskal-Wallis test was used. All variables were included for use in multivariate analyses, including hierarchical cluster analyses and projection to latent structure discriminate analysis (PLS-DA). These multivariate analysis approaches highlight groups of variables which behaved in a similar fashion and provided group segregations. Hierarchical clusters were based on a Minkowski distance matrix agglomerated using Ward’s minimum variance method. PLS-DA was performed using the SIMPLS algorithm with leave one out cross-validation, and predictive models were iteratively compared (n = 100) to those built with data randomly assigned to discriminate class data.

## Results

### PLS-DA for COHORT 1

**Figure 1:**
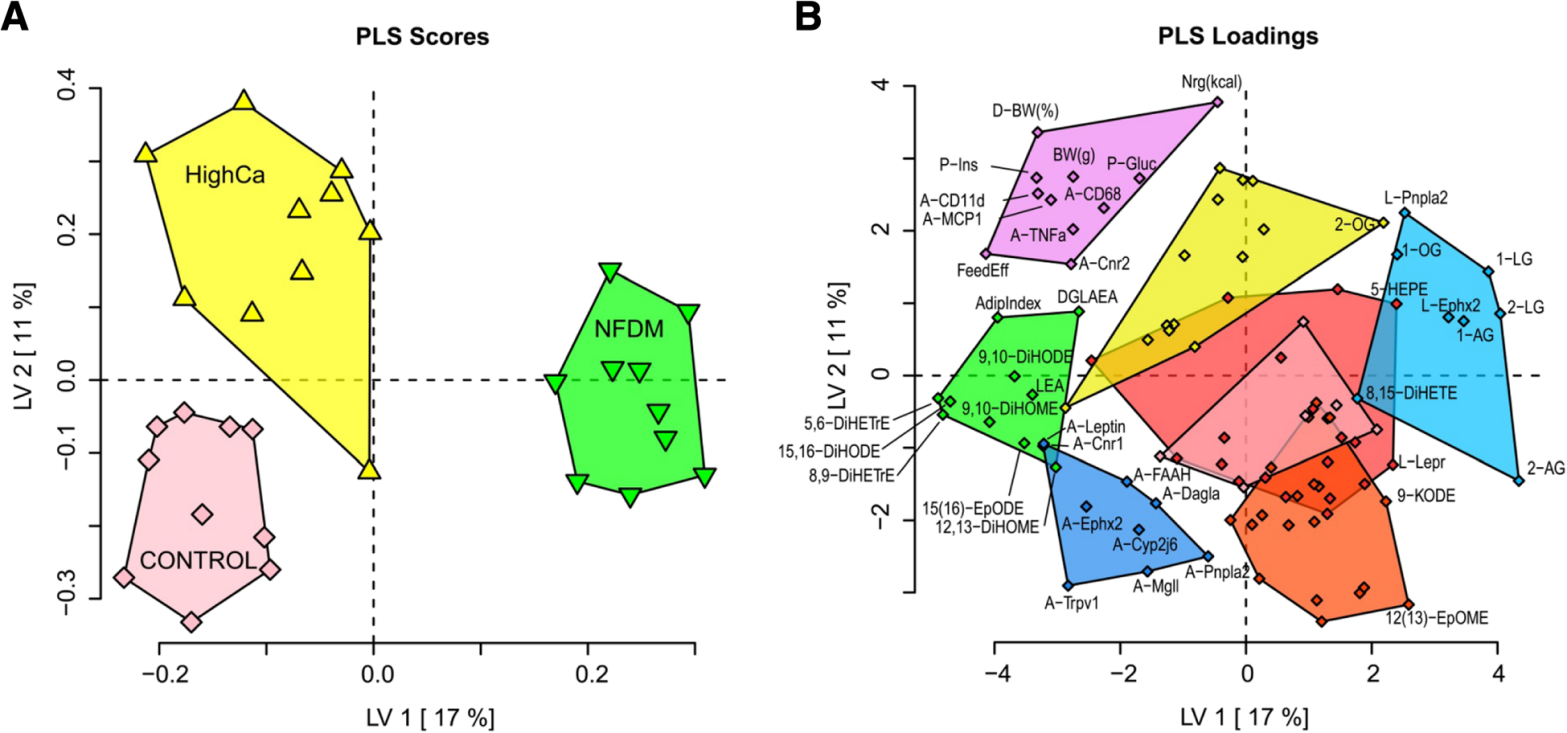
**Discriminant analysis clearly segregates dietary treatment groups in a mouse model of developing obesity (Cohort 1). A** PLS-DA model using physiological measurements, 61 plasma lipid metabolites, and mRNA relative abundance in retroperitoneal adipose tissue (A-), liver (L-), and nodose ganglia (N-) illustrates differential distribution between DIO mice fed 0.5% calcium (Control; pink), 1.5% calcium (HighCa; yellow), or 1.5% calcium + nonfat dry milk (NFDM; green). **A** plots the latent variable 1 (LV1; X axis) and 2 (LV2; Y axis) scores for each mouse, while **B** plots the variable weights in these axes. Variables were grouped and color coded based on a hierarchical cluster analysis. Selected variables in clusters and lipid classes driving group segregation are labeled (for a complete list of variables, see Additional file 3: Tables S1 and S2). Plasma fatty acid diols cluster with Adiposity Index and 18 carbon acylethanolamides (green cluster) and are reduced in NFDM-fed mice relative to Control and HighCa fed mice, along LV1. Conversely, monoacylglycerols cluster with expression of soluble epoxide hydrolase and adipose triglyceride lipase in the liver, and are elevated in NFDM-fed mice. Body weight, adiposity index, energy intake, and mRNA abundance of retroperitoneal adipose tissue inflammatory markers (purple cluster) drive the separation of HighCa fed mice from NFDM mice and controls in LV2.

To explore the impact of high-calcium and NFDM on low abundance signaling lipids during the development of obesity, a PLS-DA model was generated using plasma lipid metabolites, tissue mRNA gene expression, and phenotype parameters. Figure 1A depicts the PLS-DA scores plot output from this model showing clear separation of all 3 diet groups. Optimal separation was achieved along latent variable 1 (LV1) and LV2, and explained 28% of the variance in the data. Figure 1B is a loadings plot highlighting the features that best defined the phenotypic differences between the three diets. Variables are grouped and color-coded based on a hierarchical cluster analysis (see Methods) and significant differences of selected variables from clusters and lipid classes that drive treatment separation are depicted in Table 1 (for a complete list of plasma fatty acids analyzed, see Additional file 3: Table S1). One of the major clusters of variables driving separation along LV2 included body weight, energy intake, and inflammatory markers, with HighCa fed animals displaying increased adiposity and adipose tissue-associated inflammation and NFDM-fed animals showing decreases in these factors, relative to controls, as would be expected from our prior evaluation of these parameters in the entire cohorts using univariate statistics [17]. A discussion of this obese/inflammatory phenotype has previously been reported and will, therefore, not be a major focus of the current paper. Novel results of this model indicate two distinct lipid classes as dominant drivers of the dietary group separation (depicted along LV1): 1) diol fatty acids, products of soluble epoxide hydrolase (sEH) enzyme activity and 2) monoacylglycerols (MAGs) important to endocannabinoid biochemistry. These results are discussed in more detail below.

**Table 1:** Concentrations of selected plasma oxylipins and relative tissue mRNA abundances for oxylipin-relevant targets in mice with developing obesity

	**Cohort 1**					
	**Control**		**HighCa**		**NFDM**	
**Oxylipins (nM)**	**Mean**	**SEM**	**Mean**	**SEM**	**Mean**	**SEM**
∑*C18 Diols*						
15,16-DiHODE	1.68	0.1666^a^	1.442	0.1114^a^	0.4278	0.04809^b^
9,10-DiHOME	4.232	0.08353^a^	4.192	0.1493^a^	3.489	0.08201^b^
12,13-DiHOME	2.525	0.08735^a^	2.365	0.1339^a,b^	2.14	0.08462^b^
9,10-DiHODE	0.8443	0.01388^a^	0.865	0.02576^a^	0.7562	0.01735^b^
∑*C20 Diols*						
8,9-DiHETrE	3.995	0.1728^a^	3.345	0.1401^b^	1.536	0.07598^c^
5,6-DiHETrE	3.634	0.1948^a^	3.112	0.1064^b^	0.9325	0.06361^c^
11,12-DiHETrE	0.7705	0.05849^a^	0.7053	0.03648^a,b^	0.5728	0.03826^b^
∑*Epoxides*						
15(16)-EpODE	2.001	0.128^a^	1.899	0.1152^a^	1.419	0.0811^b^
12,13-EpOME	3.038	0.1481^a,b^	2.8	0.08468^a^	3.345	0.07784^b^
∑*Alcohols*						
5-HEPE	0.7138	0.02803^a^	0.7739	0.02809^a,b^	0.8253	0.02512^b^
**mRNA abundance (%)**						
***Adipose***						
*Cyp2j6*	100	6.8^a^	76.2	4.8^b^	82.6	5.1^a,b^
*Ephx2*	100	13.7^a^	70.4	7.9^a,b^	62.0	5.3^b^
*Leptin*	100	7.2^a^	90.3	7.5^a,b^	68.9	5.8^b^
*Trpv1*	100	8.4^a^	73.1	5.8^b^	67.5	3.2^b^
***Liver***						
*Ephx2*	100.0	8.8^a^	105.8	5.5^a^	162.8	21.5^b^

*SEM* - Standard Error of the Mean.

*n* = 10/diet treatment group.

For mRNA abundance, expression values are calculated relative to control; transcript level for CONTROL mice was considered 100%.

Data were tested for normality using D’Agostino and Pearson’s omnibus test.

To test for diet treatment differences, normal data were assessed by one-way ANOVA with Tukey post-hoc test, non-normal data were assessed by Kruskal-Wallis.

Values with different superscript letter are significantly different (P < 0.05). *ns* - not significant.

1) Plasma diol fatty acid concentrations are decreased in mice fed a high fat diet rich in NFDM. Compared to controls, decreases in several species of diol fatty acids, products of sEH enzyme activity, were detected in the plasma of NFDM fed mice (Figure 1B, green, Table 1). Endogenous substrates for sEH include cytochrome P-450 epoxygenase (CYP)-derived fatty acids (i.e. epoxyeicosatrienoic acids (EETs)), which have various bioactive properties. EETs and other epoxy fatty acids, such as the linoleate-derived EpOMEs, are readily hydrolyzed by sEH to the corresponding diols (i.e. dihydroxyeicosatrienoic acids (DHETrEs), dihydroxyoctadecamonoenoic acids (DiHOMEs), which are generally believed to be less active metabolites [29,46].

In the current investigation, circulating epoxides were unaltered by diet, while each of the four C18 diols measured were decreased in the plasma of NFDM-fed mice. HighCa did not differ significantly from controls (Table 1). Additionally, three of the four measured arachidonate-derived DHETrEs displayed significant decreases in HighCa fed mice, and even further reduction in NFDM fed mice, compared to controls. Further, the abundance of soluble epoxide hydrolase gene (*Ephx2*) mRNA, was significantly increased and decreased in the liver and RP-WAT of NFDM-fed mice, respectively, compared to controls (Table 1).

2) Several species of monoacylglycerol (MAG) are elevated in the plasma of mice fed a high fat diet rich in NFDM. According to the multivariate statistical model, plasma levels of 2-AG, a known ligand for the CB1 receptor, and other structurally-related MAGs (arachidonate-derived 1-AG, linoleate-derived 1-LG and 2-LG, and oleate-derived 1-OG and 2-OG) were contributing variables defining the separation between diet treatment groups and in particular NFDM from the other diets (Figure 1B, light blue, Table 2). PCR results indicate the expression levels of cannabinoid receptor genes *Cnr1* and *Cnr2* were down-regulated in the RP-WAT of NFDM-fed animals compared to controls (Table 2) and were not detected in liver (data not shown). Interestingly, monoacylglycerol lipase (*Mgll*), an enzyme responsible for the breakdown of 2-AG, was also down-regulated in RP-WAT of NFDM fed mice, but not significantly from controls. In contrast, plasma levels for the more potent but less abundant endogenous CB1 ligand anandamide (AEA), as well as RP-WAT mRNA abundance of the AEA catabolic enzyme fatty acid amide hydrolase (*Faah*), were not different among the three diets (AEA concentration: CONTROL, 2.467 ± 0.297; HighCa, 2.512 ± 0.242, NFDM, 2.111 ± 0.216 nM) (*Faah* relative mRNA abundance: CONTROL 100 ± 4.4, HighCa, 87.5 ± 6.1, NFDM, 83.3 ± 11.7%) (See Additional file 3: Tables S1 and S2). Furthermore, we observed decreases in two N-acyl ethanolamines (NAE) structurally related to AEA in the plasma of NFDM-fed animals, dihomogamalinoleaoylethanolamide (DGLEA) and linoleoylethanolamide (LEA) (Table 2).

**Table 2:** Concentrations of selected plasma monoacylglycerols (MAG), N-acylamides (NAE), and relative tissue mRNA abundances for endocannabinoid-relevant targets in mice with developing obesity

	**Cohort 1**					
	**Control**		**HighCa**		**NFDM**	
**Lipid analyte (nM)**	**Mean**	**SEM**	**Mean**	**SEM**	**Mean**	**SEM**
∑*MAG*						
1-AG	2.758	0.1366^a^	3.096	0.1573^a,b^	3.599	0.1214^b^
2-AG	5.718	0.3126^a^	5.553	0.3529^a^	8.841	0.3825^b^
1-LG	7.147	0.1388^a^	7.606	0.1195^a^	8.369	0.228^b^
2-LG	7.929	0.1191^a^	8.235	0.09149^a^	9.005	0.1418^b^
1-OG	7.626	0.09071^a^	8.196	0.1491^a,b^	8.287	0.2314^b^
2-OG	25.51	2.242^a^	33.74	2.376^a,b^	37.31	4.725^b^
∑*NAE*						
DGLEA	0.4023	0.04208^a,b^	0.437	0.04364^a^	0.2798	0.02968^b^
LEA	22.84	12.06^a^	7.488	3.736^a^	2.368	1.181^b^
**mRNA abundance (%)**						
***Adipose***						
*Cnr1*	100	9.8^a^	91.3	10.8^a,b^	63.8	3.7^b^
*Cnr2*	100	13.9^a^	130.3	13.0^a^	54.3	10.7^b^
*Mgll*	100	10.4^a^	71.1	5.8^b^	78.2	5.3^a,b^
***Liver***						
*Pnpla2*	100	8.4^a^	254.9	49.8^b^	234.2	42.7^a,b^

*SEM* - Standard Error of the Mean.

*n* = 10/diet treatment group.

For mRNA abundance, expression values are calculated relative to control; transcript level for CONTROL mice was considered 100%.

Data were tested for normality using D’Agostino and Pearson’s omnibus test.

To test for diet treatment differences, normal data were assessed by one-way ANOVA with Tukey post-hoc test, non-normal data were assessed by Kruskal-Wallis.

Values with different superscript letter are significantly different (P < 0.05). *ns* - not significant.

### PLS-DA for COHORT 2

**Figure 2:**
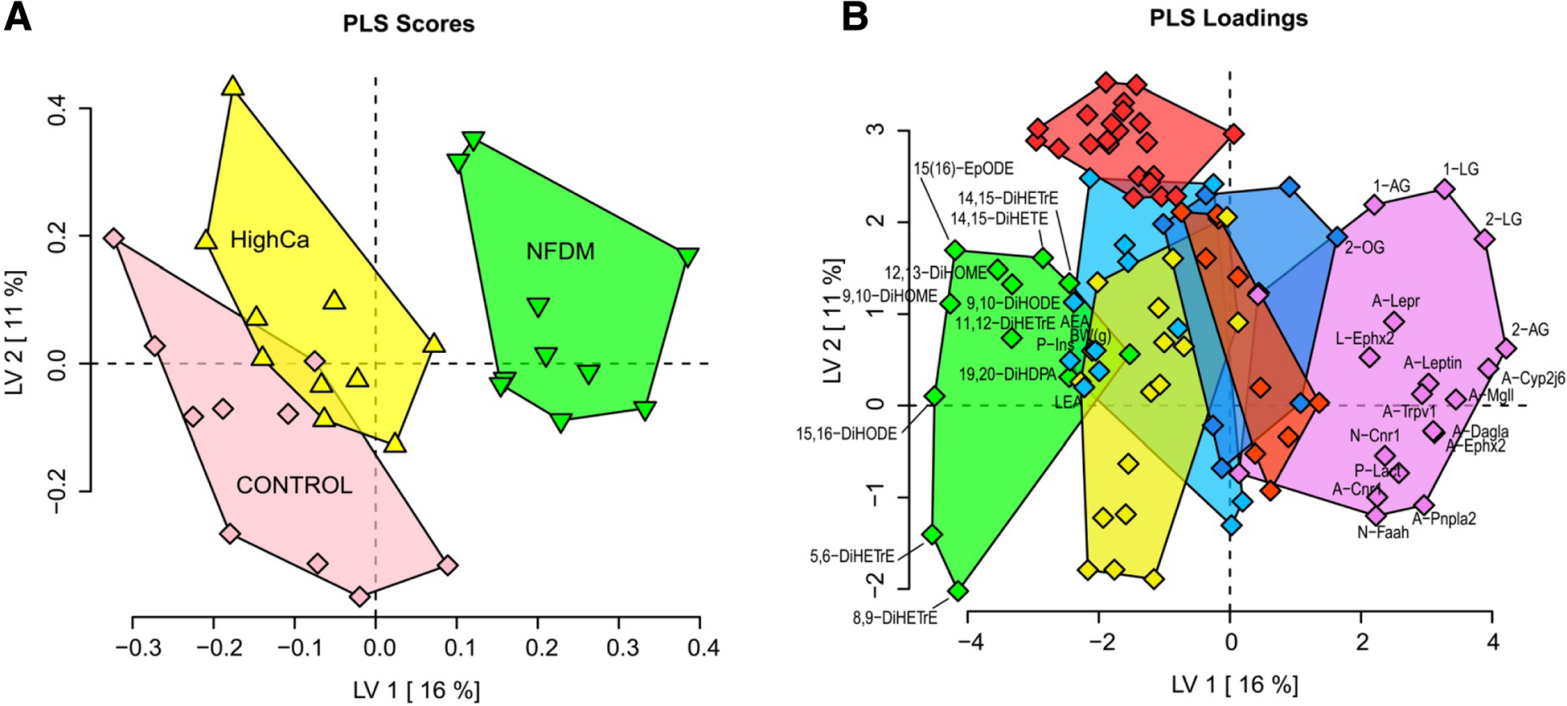
**Discriminant analysis clearly segregates dietary treatment groups in a mouse model of pre-existing obesity (Cohort 2).** In a pre-existing obesity mouse model (control DIO diet for 12 weeks), a PLS-DA model using 61 plasma lipid metabolites, physiologic measurements, and mRNA abundance in retroperitoneal adipose tissue (A-), liver (L-), and nodose ganglia (N-) illustrates differential distribution between dietary treatment groups. **A** plots the latent variable 1 (LV1; X axis) and 2 (LV2; Y axis) scores for each mouse fed 0.5% calcium (Control; pink) and 1.5% calcium (HighCa; yellow) or 1.5% calcium + nonfat dry milk (NFDM; green), while **B** plots the variable weights in these axes. Variables were grouped and color coded based on a hierarchical cluster analysis. Selected variables from clusters and lipid classes driving group segregation are labeled (for a complete list of variables, see Additional file 3: Tables S3 and S4). Plasma fatty acid diols cluster together (green cluster) and are reduced in NFDM-fed mice relative to Control and HighCa-fed mice. Conversely, monoacylglycerols cluster with a variety of adipose, liver and nodose ganglia gene expression levels (purple cluster), and are elevated in the NFDM-fed mice.

To explore the effect of high-calcium and dairy in the context of pre-existing obesity, mice in Cohort 2 underwent a pre-fattening period for 12 weeks before being randomized and placed on one of three experimental diets for an additional 8 weeks. As previously reported, pre-fattened DIO mice fed NFDM had significantly improved glucose homeostasis and markedly lower liver triglycerides, despite minimal differences in body weight, adiposity, and adipose tissue inflammation between the three diet treatment groups [17,18]. In the current investigation, the impact of high-calcium and dairy on low abundance signaling lipids in pre-fattened mice was explored using a PLS-DA model similar to Cohort 1. Figure 2A depicts the PLS-DA scores plot output from this model. Separation of the NFDM group was evident along LV1 (16% explained variance). Compared to Cohort 1, there was similar directionality in the group separations; however, controls and HighCa fed mice were not completely distinguished. Figure 2B is a loadings plot indicating selected features that defined the phenotypic differences between the three diets. Variables are color-coded by cluster analysis and significant differences of selected variables from clusters and lipid classes that drive treatment separation are depicted in Table 3. (For a complete list of plasma fatty acids analyzed, see Additional file 3: Table S3). Although body weight and adipose inflammation appear to contribute somewhat to the separation of groups, it is to a much lesser extent than as seen in Cohort 1 where body weight and inflammatory differences were much more robust, as previously reported for these conditions [18].

**Table 3:** Concentrations of selected plasma oxylipins and relative tissue mRNA abundances for oxylipin-relevant targets in mice with pre-existing obesity

	**Cohort 2**					
	**Control**		**HighCa**		**NFDM**	
**Oxylipin (nM)**	**Mean**	**SEM**	**Mean**	**SEM**	**Mean**	**SEM**
∑*C18 Diols*						
9,10-DiHOME	2.782	0.1526^a^	2.749	0.1174^a^	2.183	0.07732^b^
12,13-DiHOME	1.813	0.1115^ns^	1.752	0.07892^ns^	1.516	0.07118^ns^
9,10-DiHODE	0.04739	0.01185^a,b^	0.06502	0.0143^a^	0.01457	0.009056^b^
15,16-DiHODE	1.224	0.1558^a^	1.192	0.07191^a^	0.4152	0.04656^b^
∑*C20 Diols*						
8,9-DiHETrE	3.176	0.1345^a^	2.531	0.1684^b^	1.054	0.07053^c^
5,6-DiHETrE	2.871	0.129^a^	2.332	0.1745^a^	0.7654	0.157^b^
11,12-DiHETrE	0.753	0.04288^a^	0.7909	0.0363^a^	0.6191	0.03119^b^
14,15-DiHETrE	0.9577	0.03963^a,b^	1.05	0.0584^a^	0.88	0.06521^b^
14,15-DiHETE	0.9577	0.03963^a,b^	1.05	0.0584^a^	0.88	0.06521^b^
17,18-DiHETE	1.691	0.08384^a,b^	1.883	0.07553^a^	1.56	0.03399^b^
∑*C22 Diols*						
19,20-DiHDoPE	2.711	0.2254^a,b^	3.41	0.2376^a^	2.242	0.2042^b^
∑*Epoxides*						
15(16)-EpODE	1.524	0.1816^a^	1.501	0.1288^a^	0.9132	0.07219^b^
**mRNA abundance (%)**						
***Adipose***						
*Cyp2j6*	100	9.5^a^	123.2	14.8^a^	218.1	29.8^b^
*Ephx2*	100	15.7^a,b^	79.55	13.0^a^	144.6	17.3^b^
*Leptin*	100	151.3^ns^	48.84	59.1^ns^	15.4	18.7^ns^
*Trpv1*	100	9.3^ns^	99.7	9.1^ns^	139.5	19.7^ns^
***Liver***						
*Ephx2*	100	6.1^ns^	98.45	7.0^ns^	123.0	10.9^ns^

*SEM* - Standard Error of the Mean.

*n* = 10/diet treatment group; For mRNA abundance, expression values are calculated relative to control; transcript level for CONTROL mice was considered 100%.

Data were tested for normality using D’Agostino and Pearson’s omnibus test.

To test for diet treatment differences, normal data were assessed by one-way ANOVA with Tukey post-hoc test, non-normal data were assessed by Kruskal-Wallis.

Values with different superscript letter are significantly different (P < 0.05). *ns* - not significant.

Similar to Cohort 1, the lipid variables contributing most to the separation between diets were again diol fatty acids and MAGs:

1) Plasma diol fatty acid concentrations are decreased in NFDM fed mice with pre-existing obesity. Compared to controls, there was a trend in decreasing C18 diols; however, only 9,10-DiHOME and 15,16-DiHODE reached significance (Table 3). We observed a significant decrease in three of the four measured C20 arachidonate-derived vicinal diols in NFDM fed animals, while HighCa fed animals were not different from controls. Unlike Cohort 1, mRNA abundance for *Ephx2* in both the liver and adipose was unaffected by diet in Cohort 2.

2) MAG concentrations are elevated in the plasma of NFDM fed animals with pre-existing obesity. The increases in plasma MAGs seen in this cohort were much more modest (10-20%) than the increases seen in Cohort 1, with only 2-AG, 1-LG and 2-LG reaching statistical significance (Table 4). Nevertheless, as a group these metabolites were important discriminating variables distinguishing the NFDM group from the Control and HighCa groups. PCR results in RP-WAT did not show significant differences in mRNA abundances for *Cnr1* or *Cnr2* (Table 4) or *Faah* (Additional file 3: Table S4); however, *Mgll* was significantly increased in NFDM fed animals, compared to controls. Plasma levels of all NAEs measured did not differ among the three diets.

**Table 4:** Concentrations of selected plasma monoacylglycerols (MAG), N-acylamides (NAE), and relative tissue mRNA abundances for endocannabinoid-relevant targets in mice with pre-existing obesity

	**Cohort 2**					
	**Control**		**HighCa**		**NFDM**	
**Lipid analyte (nM)**	**Mean**	**SEM**	**Mean**	**SEM**	**Mean**	**SEM**
∑*MAG*						
1-AG	2.922	0.1194^ns^	3.132	0.2386^ns^	3.437	0.2354^ns^
2-AG	5.44	0.07841^a^	5.437	0.06266^a^	5.919	0.1294^b^
1-LG	4.964	0.222^a^	5.295	0.1451^a,b^	6.067	0.291^b^
2-LG	8.196	0.1433^a^	8.293	0.09063^a^	9.149	0.1696^b^
1-OG	7.399	0.1517^ns^	7.829	0.1807^ns^	7.828	0.2093^ns^
2-OG	8.197	0.1308^ns^	8.553	0.1325^ns^	8.665	0.1947^ns^
∑*NAE*						
AEA	2.093	0.1251^ns^	2.81	0.2002^ns^	1.744	0.1747^ns^
LEA	20.92	1.406^ns^	22.21	1.996^ns^	16.22	1.885^ns^
**mRNA abundance (%)**						
***Adipose***						
*Mgll*	100	12.0^a^	73.5	10.9^a^	162.9	19.3^b^
*Cnr1*	100	15.4^ns^	91.2	13.7^ns^	130.2	20.6^ns^
*Cnr2*	100	15.9^ns^	140.8	18.7^ns^	85.08	14.1^ns^
*Pnpla2*	100	13.5^a,b^	68.7	8.8^a^	132.9	17.7^b^
***Nodose***						
*Cnr1*	100	10.2^a,b^	88.7	6.5^a^	117.6	7.1^b^

*SEM* - Standard Error of the Mean.

*n* = 10/diet treatment group.

For mRNA abundance, expression values are calculated relative to control; transcript level for CONTROL mice was considered 100%.

Data were tested for normality using D’Agostino and Pearson’s omnibus test.

To test for diet treatment differences, normal data were assessed by one-way ANOVA with Tukey post-hoc test, non-normal data were assessed by Kruskal-Wallis.

Values with different superscript letter are significantly different (P < 0.05). *ns* - not significant.

## Discussion

A diet rich in dairy has been associated with improvements in body composition, metabolic disease risk markers and inflammatory status; however, the mechanisms by which dairy exerts its effects are still being elucidated. In the current study, we identified unique profiles of oxylipins and endocannabinoids in the plasma of DIO mice fed a diet rich in NFDM. As previously reported, these NFDM fed animals displayed decreased adiposity, markedly reduced steatosis, and lower adipose inflammation in comparison to control/high-calcium, soy protein based high fat diets [17,18]. The results described herein highlight that these phenotypic differences may be associated with alterations in circulating bioactive lipids.

Biosynthesis of oxylipins is catalyzed by cyclo-oxygenase (COX), lipoxygenase (LOX), and cytochrome p450 (CYP) enzymes [29]. The bioactive products of these pathways have broad cellular effects on vascular [47], hepatic [48], and adipose physiology [32], and, depending on the specific metabolites involved, can exert both pro-inflammatory and anti-inflammatory effects. In the current investigation, dietary treatment resulted in distinct changes to patterns of plasma lipids emanating from the CYP epoxygenase pathways, but not LOX and COX pathways. Specifically, mice fed a NFDM based diet displayed decreases in several species of diol fatty acids, products of soluble epoxide hydrolase (sEH) enzyme activity, compared to controls or mice fed high calcium alone. These effects were observed in two separate cohorts of mice, each with disparate weight outcomes in response to NFDM and HighCa, suggesting that changes in diol biochemistry were unique to the NFDM diet and weight/adiposity-independent.

The major CYP-derived products include epoxide fatty acids (i.e. arachidonate-derived epoxyeicosatrienoic acids (EETs)). Recent evidence suggests EETS have beneficial effects on adipose and liver insulin sensitivity through the reduction of ER stress [49,50], and that DHETs, products of EET hydrolysis by sHE, exert their own opposing activity (DHETs increase ER stress and attenuate insulin sensitivity). Our findings are consistent with the role for epoxide/sEH/diol activity in mediating inflammation and insulin signaling. While epoxides were similar across dietary treatment groups, decreased plasma diols observed in the plasma of NFDM fed mice were associated with this group’s improved glucose tolerance, dampened inflammation, and reduced liver steatosis, both in developing obesity (Cohort 1) [17] and pre-existing obesity (Cohort 2) [18].

The mechanism by which NFDM decreases plasma diols is unclear, but could result from direct dietary effects on relevant biochemical pathways and substrates flowing through the CYP epoxygenase/sEH pathways, or proceed secondary to metabolic and inflammatory phenotypes associated with these diets. While fatty acid composition was the same for all experimental diets, other components of dairy, such as dairy protein, may alter appetite-regulating hormones [24], thereby decreasing total food intake and overall total fatty acid substrates. However, cumulative energy intake was not a discriminating variable in the PLS-DA model, did not cluster with diol fatty acids, and was not reduced in NFDM-fed mice [17,18]. Alternatively, diet-associated signals or the attenuated obesity phenotype in NFDM mice could have reduced tissue sEH expression and/or activity. It has been previously reported that sEH protein is increased in adipose and liver of DIO mice and obese humans [50], presumably decreasing the beneficial activity of epoxides. In the current investigation, *Epxh2* (sEH) mRNA expression was elevated in the liver and reduced in the adipose of NFDM fed animals during developing obesity, but these changes were not apparent in the presence of pre-existing obesity. However, changes in sEH activity have been reported despite no changes in mRNA expression [51]. Furthermore, it is unclear how plasma pools of oxylipins reflect tissue specific enzyme activity, and whether paracrine activity is more important to the phenotype observed. Future studies will be needed to explore if NFDM alters tissue-specific diol production and sEH activity or protein abundance. Nevertheless, we have demonstrated for the first time that circulating oxylipins are altered in DIO mice fed a diet rich in NFDM, a diet that improves inflammatory status and glucose homeostasis.

In addition to possible effects of oxylipin changes in supporting NFDM-associated phenotypes, the endocannabinoid system and structurally-related analogs were considered due to their deep involvement in the control of food intake and energy homeostasis [52]. In this study, several species of MAG, including 2-AG, increased in the plasma of DIO mice fed NFDM compared to the Control or HighCa diets, under the conditions of both developing obesity and pre-existing obesity. 2-AG has been shown to be a more efficacious cannabinoid receptor agonist than AEA [53], while other 2-MAGS have been shown to potentiate the activity of eCBs (i.e. 2-LG) [54] or have GPR119 agonist activity (i.e. 2-OG) [55]. Others have reported that 2-AG is increased in peripheral tissues of animals fed a high-fat diet (HFD) [56,57], and circulating 2-AG levels positively correlate with BMI and intra-abdominal adiposity in humans [58,59], although this observation has not been universal [40]. An association between eCB tone and obesity has been hypothesized to exacerbate obesity-related dysmetabolic conditions by increasing food intake and liver fatty acid synthesis through cannabinoid type-1 (CB1) receptor signaling [36,52,60]. However, in the current study, increases in circulating 2-AG levels in NFDM fed animals were concurrent with markedly reduced liver steatosis, even in Cohort 2 where body weight differences from Control animals were minimal [17,18]. Similar findings were reported in a mouse model where tissue levels of 2-AG were artificially elevated by utilizing a monoglyceride lipase (*Mgl*) global knockout, the primary degrading enzyme for 2-AG [61]. These animals did not demonstrate the expected hyperphagia and increased weight gain when fed a high fat diet and exhibited improved insulin sensitivity and considerably less liver steatosis, similar to NFDM-fed mice seen herein. The authors also reported desensitization in liver CB1 receptor signaling in *Mgl* knockouts. Although liver CB1 and CB2 mRNA were not detected in the present study, we did observe a significant decrease in CB1 and CB2 receptor mRNA in the RP WAT of NFDM fed animals, at least in Cohort 1 where increases in 2-AG were much more robust. Decreased adipose CB1 receptor gene expression was also associated with increased systemic 2-AG concentrations in viscerally-obese subjects [58]. While speculative, this raises the possibility that, in NFDM-fed mice, negative feedback regulation of cannabinoid receptor signaling by elevated 2-AG contributed to the lean phenotype.

In addition to 2-AG, several other species of MAG were elevated in the plasma of NFDM fed mice, prompting us to quantify the mRNA abundances of enzymes involved in synthesis (fatty acid liberation via lipolysis) and degradation of these metabolites in RP-WAT, liver, and nodose ganglia (containing vagal afferent neurons), sites that represent tissues responsive to endocannabinoid actions. In our multivariate model, mRNA abundances for these targets weakly contributed to the separation of treatment groups. However, it is acknowledged that mRNA expression may not reflect protein levels or enzyme activity. Alternatively, elevations of plasma 2-AG observed here may be a regulatory response to anti-inflammatory signals stemming from the NFDM diet. Endocannabinoids, including 2-AG, exert immunomodulatory effects across many different cell types and tissues [62], and it has been reported that plasma 2-AG is decreased under acute inflammatory exposure [63].

Furthermore, enzyme activity and eCB signaling in tissue sites not sampled herein may contribute to the plasma pool of metabolites or drive the observed phenotype. For instance, intestinal eCB signaling influences food intake via vagal afferents [64-66] and models of intestinal inflammation increase eCB tone [67,68]. Also, cross-talk between the eCB system and the gut microbiome can alter adipose physiology [69] and recent studies demonstrated that administration of *Akkermansia muciniphila*, a bacterium associated with an improved metabolic profile, increased intestinal levels of the monoglycerols 2-AG, 2-OG and 2-PG [70]. Intriguingly, we have detected significant differences in the microbiome composition of the mice fed NFDM compared to the other dietary groups [71], raising the possibility that intestinal alterations are responsible for elevations in systemic 2-AG and possibly other metabolites.

It is important to address whether the distinct pattern of lipid metabolites seen here is due to a direct dietary effect or a secondary consequence of the phenotype. Studies addressing dietary influences on oxylipins and eCBs primarily focus on amount and composition of dietary fat [38,72,73]. In the current study, the source and macronutrient content of fat were identical across treatment groups and was presumably not a driver in the treatment associated phenotypes. Furthermore, high-calcium alone is not sufficient to exert the effects seen here, since HighCa and NFDM fed animals, whom both received 1.5% calcium in their diets, discriminated independently from one another in our multivariate models. In future studies it will be interesting to determine if NFDM effects on lipid and enzyme phenotypes, reported herein, are modulated by altering dietary Ca intake. Regardless, there is evidence that the lipidomic patterns seen here are influenced by diet and are not simply a consequence of a lean or obese phenotype. First, lipid metabolites that best discriminate treatment differences in our multivariate model cluster independently from body weight, adiposity, adipose inflammatory markers, and food intake. Second, the pattern of discriminating factors observed in our model was observed in both Cohort 1 (developing obesity) and Cohort 2 (pre-existing obesity) studies despite markedly different feeding paradigms and body weight outcomes.

## Conclusion

In summary, this study illustrates that differences in bioactive lipid profiles are associated with metabolic and inflammatory phenotypes seen in NFDM mice, which include reduced weight gain, improved glucose homeostasis, avoidance of steatosis and dampened inflammatory markers. NFDM fed mice showed a decrease in plasma diols which is consistent with a possible role for epoxide/diol balance in regulating insulin sensitivity and inflammation. Furthermore, increases in circulating 2-AG seen in NFDM may constitute an adaptive mechanism to increase food intake in response to other dietary regulatory inputs that drive the lean phenotype and reduced feed efficiency observed in these mice. Future studies are necessary to reveal the exact mechanisms by which these lipidomic shifts occur, and to elucidate whether these lipid metabolites participate in the metabolic effects of dairy-rich diets.

## Competing interests

The authors declare that they have no competing interests.

## Authors’ contributions

TND, AHK, APT, SHA conducted animal model research and/or sample analyses; TND, AHK, JWN conducted statistical analysis; TND, AHK, JWN, SHA interpreted results; and TND drafted the manuscript with edits from all authors. All authors read and approved the final manuscript.

## Supplementary Material

Additional file 1: Table S5 UPLC/MS-MS parameters of oxylipins and endocannabinoids measured in plasma. Click here for file

Additional file 2: Figure S1 Representative chromatograms of 2-AG and 1-AG resolution from a single plasma sample and low level calibration standard. Click here for file

Additional file 3: Table S1 Mean concentrations (nM) of plasma fatty acids in mice with developing obesity. **Table S2.** Relative mRNA abundances and physiological measurments in mice with developing obesity. **Table S3.** Mean concentrations (nM) of plasma fatty acids in mice with pre-existing obesity. **Table S4.** Relative mRNA abundances and physiological measurments in mice with pre-exisiting obesity. Click here for file
