# Supplementary material for: A diet containing a nonfat dry milk matrix significantly alters systemic oxylipins and the endocannabinoid 2-arachidonoylglycerol (2-AG) in diet-induced obese mice
Source: Nutr Metab (Lond). 2014 May 30;11:24. doi: 10.1186/1743-7075-11-24 (PMC4068977; doi:10.1186/1743-7075-11-24)
Supplement: Additional file 1: Table S5 — UPLC/MS-MS parameters of oxylipins and endocannabinoids measured in plasma. [file 1743-7075-11-24-S1.docx]

**Additional file 1: Table S5. UPLC/MS-MS parameters of oxylipins and endocannabinoids measured in plasma**

| **Compound** | **tR (min)** | **Precursor (m/z)** | **Product (m/z)** | **DCP (V)** | **CE (V)** | **IS/ Surrogate** |
| --- | --- | --- | --- | --- | --- | --- |
| PHAU | 3.34 | 249.2 | 130.1 | -45 | -20 |  |
| 20-carboxy-LTB4 | 3.58 | 365.3 | 347.2 | -70 | -25 | d4 6-keto PGF1a |
| Resolvin E1 | 3.71 | 349.3 | 195.2 | -70 | -25 | d4 6-keto PGF1a |
| 6-keto-PGF1a | 3.71 | 369.3 | 163.1 | -70 | -40 | d4 6-keto PGF1a |
| d4 6-keto PGF1a | 3.71 | 373.3 | 167.1 | -60 | -40 | PHAU |
| 20-hydroxy-LTB4 | 3.8 | 351.3 | 195.2 | -65 | -25 | d4 6-keto PGF1a |
| PGE3 | 4.53 | 349.3 | 269.2 | -45 | -22 | d4-PGD2a |
| d4-TXB2 | 4.52 | 373.3 | 173.1 | -50 | -25 | PHAU |
| TXB2 | 4.54 | 369.3 | 169.1 | -50 | -25 | d4-TXB2 |
| 9,12,13-TriHOME | 5.00 | 329.2 | 211.2 | -70 | -32 | d4-PGF2a |
| d4-PGF2a | 5.06 | 357.3 | 197.2 | -65 | -35 | PHAU |
| PGF2a / F2-IsoPs | 5.07 | 353.3 | 193.2 | -65 | -35 | d4-PGF2a |
| 9,10,13-TriHOME | 5.11 | 329.2 | 171.1 | -70 | -30 | d4-PGF2a |
| PGE2 | 5.28 | 351.3 | 271.2 | -35 | -25 | d4-PGD2a |
| PGE1 | 5.36 | 353.3 | 317.2 | -50 | -22 | d4-PGD2a |
| d4-PGD2 | 5.49 | 355.3 | 275.2 | -45 | -26 | PHAU |
| PGD2 | 5.62 | 351.3 | 271.2 | -35 | -25 | d4-PGD2a |
| Resolvin D1 | 6.12 | 375.3 | 121.1 | -50 | -40 | d4-PGF2a |
| 11,12,15 THET | 6.03 | 353.3 | 167.1 | -60 | -30 | d4-PGF2a |
| Lipoxin A4 | 6.22 | 351.3 | 217.2 | -50 | -26 | d4-PGF2a |

**Supplemental Table 5. UPLC/MS-MS parameters of oxylipins and endocannabinoids measured in plasma (continued)**

| **Compound** | **tR (min)** | **Precursor (m/z)** | **Product (m/z)** | **DCP (V)** | **CE (V)** | **IS/ Surrogate** |
| --- | --- | --- | --- | --- | --- | --- |
| PGJ2/ δ 12-PGJ2 | 7.13 | 333.2 | 233.2 | -52 | -14 | d4-PGF2a |
| PGB2 | 7.11 | 333.3 | 235.2/175.1 | -74 | -28 | d4-PGF2a |
| LTB5 | 7.35 | 333.3 | 195.2 | -70 | -20 | d4-PGF2a |
| DiHETHF | 7.41 | 353.3 | 127.1 | -55 | -35 | d11-14,15-DiHETrE |
| 15,16-DiHODE | 7.69 | 311.2 | 235.2 | -65 | -22 | d11-14,15-DiHETrE |
| 12,13-DiHODE | 7.79 | 311.2 | 183.2 | -70 | -30 | d11-14,15-DiHETrE |
| 8,15-DiHETE | 7.8 | 335.3 | 235.2 | -65 | -22 | d11-14,15-DiHETrE |
| Hepoxilin A3 | 7.81 | 335.2 | 171.1 | -85 | -20 | d11-14,15-DiHETrE |
| 9,10-DiHODE | 7.83 | 311.2 | 201.2 | -65 | -30 | d11-14,15-DiHETrE |
| d3-LTE4 | 7.88 | 441.4 | 336.3 | -80 | -30 | CUDA |
| LTE4 | 7.91 | 438.4 | 333.3 | -60 | -25 | d3-LTE4 |
| 17,18-DiHETE | 8.08 | 335.3 | 247.2 | -60 | -25 | d11-14,15-DiHETrE |
| 5,15-DiHETE | 8.15 | 335.3 | 173.1 | -45 | -21 | d11-14,15-DiHETrE |
| 6-trans-LTB4 | 8.26 | 335.3 | 195.2 | -70 | -21 | d4-LTB4 |
| 14,15-DiHETE | 8.42 | 335.3 | 207.2 | -55 | -25 | d11-14,15-DiHETrE |
| CUDA | 8.47 | 339.4 | 214.2 | -65 | -35 |  |
| d4-LTB4 | 8.49 | 339.3 | 163.1 | -70 | -38 | CUDA |
| LTB4 | 8.6 | 335.3 | 195.2 | -70 | -21 | d4-LTB4 |
| 12,13-DiHOME | 8.81 | 313.3 | 183.2 | -70 | -30 | d11-14,15-DiHETrE |
| 10,11-DHHep | 8.9 | 301.2 | 283.2 | -70 | -30 | CUDA |
| 9,10-DiHOME | 9.23 | 313.3 | 201.2 | -70 | -30 | d11-14,15-DiHETrE |
| d11-14,15-DiHETrE | 9.39 | 348.4 | 207.2 | -64 | -28 | CUDA |
| LTD4 | 9.41 | 495.45 | 177.1 | -70 | -25 | d3-LTE4 |

**Supplemental Table 5. UPLC/MS-MS parameters of oxylipins and endocannabinoids measured in plasma (continued)**

| **Compound** | **tR (min)** | **Precursor (m/z)** | **Product (m/z)** | **DCP (V)** | **CE (V)** | **IS/ Surrogate** |
| --- | --- | --- | --- | --- | --- | --- |
| 19,20-DiHDoPE | 9.44 | 361.3 | 273.2 | -74 | -24 | d11-14,15-DiHETrE |
| 14,15-DiHETrE | 9.48 | 337.3 | 207.2 | -65 | -25 | d11-14,15-DiHETrE |
| 11,12-DiHETrE | 10.06 | 337.3 | 167.1 | -60 | -27 | d11-14,15-DiHETrE |
| 9-HOTE | 10.32 | 293.2 | 171.1 | -65 | -22 | d4-9-HODE |
| 12(13)-Ep-9-KODE | 10.33 | 309.2 | 291.2 | -65 | -20 | d4-9-HODE |
| 13-HOTE | 10.44 | 293.2 | 195.2 | -65 | -25 | d4-9-HODE |
| 8,9-DiHETrE | 10.61 | 337.3 | 127.1 | -55 | -30 | d11-14,15-DiHETrE |
| 15-deoxy PGJ2 | 10.68 | 315.2 | 271.2 | -60 | -20 | d11-14,15-DiHETrE |
| d6-20-HETE | 10.86 | 325.3 | 281.2 | -70 | -25 | CUDA |
| 15-HEPE | 10.88 | 317.2 | 219.2 | -60 | -20 | d8-12-HETE |
| 20-HETE | 10.91 | 319.2 | 275.2 | -65 | -24 | d6-20-HETE |
| 12-HEPE | 11.23 | 317.2 | 179.1 | -60 | -20 | d8-12-HETE |
| 5,6-DiHETrE | 11.38 | 337.3 | 145.1 | -70 | -25 | d11-14,15-DiHETrE |
| 13-HODE | 11.84 | 295.2 | 195.2 | -65 | -25 | d4-9-HODE |
| 5-HEPE | 11.84 | 317.2 | 115.1 | -60 | -20 | d8-5-HETE |
| d4-9-HODE | 11.89 | 299.2 | 172.1 | -70 | -26 | CUDA |
| 9-HODE | 11.95 | 295.2 | 171.1 | -60 | -25 | d4-9-HODE |
| 15(16)-EpODE | 12.02 | 293.2 | 275.2 | -60 | -20 | d4-12(13)-EpOME |
| 17(18)-EpETE | 12.11 | 317.2 | 259.2 | -55 | -15 | d4-12(13)-EpOME |
| 15-HETE | 12.13 | 319.2 | 219.2 | -70 | -16 | d8-12-HETE |
| 13-KODE | 12.21 | 293.2 | 179.1 | -70 | -26 | d4-9-HODE |
| 15-HpETE | 12.21 | 335.2 | 113.1 | -58 | -20 | d8-12-HETE |
| 9(10)-EpODE | 12.22 | 293.2 | 275.2 | -60 | -20 | d4-12(13)-EpOME |
| 17-HDoHE | 12.22 | 343.3 | 281.2 | -45 | -20 | d8-12-HETE |
| 13-HpODE | 12.41 | 311.2 | 179.1 | -40 | -20 | d4-9-HODE |

**Supplemental Table 5. UPLC/MS-MS parameters of oxylipins and endocannabinoids measured in plasma (continued)**

| **Compound** | **tR (min)** | **Precursor (m/z)** | **Product (m/z)** | **DCP (V)** | **CE (V)** | **IS/ Surrogate** |
| --- | --- | --- | --- | --- | --- | --- |
| 12(13)-EpODE | 12.42 | 293.2 | 183.2 | -60 | -25 | d4-12(13)-EpOME |
| 15-KETE | 12.48 | 317.2 | 273.2 | -65 | -20 | d8-12-HETE |
| 11-HETE | 12.57 | 319.2 | 167.1 | -55 | -15 | d8-12-HETE |
| 14(15)-EpETE | 12.59 | 317.2 | 247.2 | -45 | -15 | d4-12(13)-EpOME |
| 9-KODE | 12.66 | 293.2 | 185.2 | -70 | -30 | d4-9-HODE |
| d8-12-HETE | 12.71 | 327.2 | 184.2 | -60 | -21 | CUDA |
| 9-HpODE | 12.79 | 311.2 | 185.2 | -40 | -20 | d4-9-HODE |
| 12-HETE | 12.82 | 319.2 | 179.1 | -60 | -21 | d8-12-HETE |
| 8-HETE | 12.96 | 319.2 | 155.1 | -55 | -21 | d8-12-HETE |
| 12-HpETE | 12.98 | 335.2 | 153.1 | -58 | -20 | d8-12-HETE |
| 15-HETrE | 13.02 | 321.2 | 221.2 | -65 | -24 | d8-12-HETE |
| 12-KETE | 13.07 | 317.2 | 273.2 | -65 | -20 | d8-12-HETE |
| 9-HETE | 13.16 | 319.2 | 167.1 | -55 | -15 | d8-12-HETE |
| d8-5-HETE | 13.4 | 327.2 | 116.1 | -55 | -22 | CUDA |
| 19(20)-EpDoPE | 13.46 | 343.3 | 281.2 | -45 | -20 | d4-12(13)-EpOME |
| 5-HETE | 13.5 | 319.2 | 257.2 / 115.1 | -50 | -20 | d8-5-HETE |
| d4-12(13)-EpOME | 13.57 | 299.2 | 198.1 | -65 | -25 | CUDA |
| 12(13)-EpOME | 13.65 | 295.2 | 195.1 | -65 | -25 | d4-12(13)-EpOME |
| 14(15)-EpETrE | 13.73 | 319.2 | 219.2 | -70 | -16 | d4-12(13)-EpOME |
| 16(17)-EpDoPE | 13.84 | 343.5 | 273.5 | -55 | -15 | d4-12(13)-EpOME |
| 9(10)-EpOME | 13.88 | 295.2 | 171.1 | -60 | -25 | d4-12(13)-EpOME |
| 5-HpETE | 14.06 | 335.2 | 155.1 | -58 | -20 | d8-12-HETE |
| 5-KETE | 14.16 | 317.2 | 203.2 | -70 | -25 | d8-5-HETE |
| 11(12)-EpETrE | 14.2 | 319.2 | 167.1 | -55 | -15 | d4-12(13)-EpOME |
| 8(9)-EpETrE | 14.4 | 319.2 | 155.1/ 167.1 | -55 | -15 | d4-12(13)-EpOME |

Supplemental Table 5. UPLC/MS-MS parameters of oxylipins and endocannabinoids measured in plasma (continued)

| **Compound** | **Abbreviation** | **tR (min)** | **Precursor  Ion (m/z)** | **Product**  **Ion (m/z)** | **DCP (V)** | **CE (V)** | **IS/ Surrogate** |
| --- | --- | --- | --- | --- | --- | --- | --- |
| Prostaglandin D2-EA*^a^* | PGD2-EA | 2.90 | 378.3 | 62.1 | 55 | 40 | d4-PGF2a-EA |
| Prostaglandin D2-F2a-EA | d4-PGF2a-EA | 3.24 | 384.3 | 62.1 | 55 | 41 | CUDA |
| Prostaglandin F2a-EA | PGF2a-EA | 3.25 | 380.3 | 62.1 | 58 | 38 | d4-PGF2a-EA |
| Prostaglandin E2-EA | PGE2-EA | 3.26 | 378.3 | 62.1 | 58 | 38 | d4-PGF2a-EA |
| Prostaglandin F2a-1G*^b^* | PGF2a-1G | 3.04 | 411.3 | 301.2 | 55 | 19 | d4-PGF2a-EA |
| Prostaglandin E2-1G | PGE2-1G | 3.07 | 409.3 | 317.2 | 55 | 19 | d4-PGF2a-EA |
| 1-Cyclohexyl-urido-3-dodecanoic acid | CUDA | 4.98 | 341.3 | 216.2 | 58 | 25 | --- |
| α-linolenoyl-EA | ALEA | 6.03 | 322.2 | 62.1 | 72 | 32 | d8-A-EA |
| Docosahexenoyl-EA | DoHexEA | 6.42 | 372.3 | 62.1 | 61 | 36 | d8-A-EA |
| d8-Arachidonoyl-EA | d8-AEA | 6.54 | 356.3 | 63.1 | 60 | 30 | CUDA |
| Arachidonoyl-EA | AEA | 6.59 | 348.3 | 62.1 | 65 | 33 | d8-A-EA |
| Linoleoyl-EA | LEA | 6.66 | 324.2 | 62.1 | 72 | 31 | d8-A-EA |
| d5-2-Arachidonoyl-G | d5-2-AG | 6.92 | 384.3 | 287.2 | 63 | 19 | CUDA |
| 2-Arachidonoyl-G | 2-AG | 6.94 | 379.3 | 287.2 | 53 | 19 | d5-2-AG |
| Dihomo-γ-linoleoyl-EA | DGLEA | 7.02 | 350.3 | 62.1 | 65 | 36 | d8-A-EA |
| 1-Arachidonoyl-G | 1-AG | 7.10 | 379.3 | 287.2 | 53 | 19 | d5-2-AG |
| 2-Linoleoyl-G | 2-LG | 7.11 | 355.3 | 263.2 | 52 | 18 | d5-2-AG |
| d4-Palmitoyl-EA | d4-PEA | 7.31 | 304.2 | 62.1 | 80 | 35 | CUDA |
| 1-Linoleoyl-G | 1-LG | 7.32 | 355.3 | 263.2 | 52 | 18 | d5-2-AG |
| Palmitoyl-EA | PEA | 7.33 | 300.2 | 62.1 | 80 | 31 | d4-P-EA |
| Oleoyl-EA | OEA | 7.60 | 326.2 | 62.1 | 80 | 32 | d4-P-EA |
| 2-Oleoyl-G | 2-OG | 8.20 | 357.3 | 265.2 | 52 | 18 | d5-2-AG |
| 1-Oleoyl-G | 1-OG | 8.86 | 357.3 | 265.2 | 52 | 18 | d5-2-AG |
| Stearoyl-EA | SEA | 8.97 | 328.2 | 62.1 | 80 | 35 | d4-P-EA |

*a* - EA, ethanolamide; *b* - G, glycerol
