# Supplementary material for: A diet containing a nonfat dry milk matrix significantly alters systemic oxylipins and the endocannabinoid 2-arachidonoylglycerol (2-AG) in diet-induced obese mice
Source: Nutr Metab (Lond). 2014 May 30;11:24. doi: 10.1186/1743-7075-11-24 (PMC4068977; doi:10.1186/1743-7075-11-24)
Supplement: Additional file 2: Figure S1 — Representative chromatograms of 2-AG and 1-AG resolution from a single plasma sample and low level calibration standard. [file 1743-7075-11-24-S2.docx]

**Supplemental Figure 1.** Representative chromatograms of 2-AG and 1-AG resolution from a single plasma sample and low level calibration standard
